# Absence of high-level vancomycin resistance in enterococci isolated from meat-processing facilities.

**DOI:** 10.3201/eid0706.010620

**Published:** 2001

**Authors:** P W Bodnaruk, P J Krakar, R B Tompkin

**Affiliations:** ConAgra Refrigerated Prepared Foods, Downers Grove, Illinois 60515, USA.

## Abstract

Enterococci isolated from packaging areas of meat-processing facilities that produce ready-to-eat meat products were examined for high-level vancomycin resistance. A total of 406 enterococci isolates from the plants' packaging areas were examined for vancomycin resistance. High-level vancomycin resistance was not demonstrated in any enterococci isolated from 12 meat-processing plants.


### Absence of High-Level Vancomycin Resistance in Enterococci Isolated from Meat-Processing Facilities

**Peter W. Bodnaruk, Patrick J. Krakar, and R. Bruce Tompkin**
ConAgra Refrigerated Prepared Foods, Downers Grove, Illinois, USA

Enterococci isolated from packaging areas of meat-processing facilities that produce ready-to-eat meat products were examined for high-level vancomycin resistance. A total of 406 enterococci isolates from the plants' packaging areas were examined for vancomycin resistance. High-level vancomycin resistance was not demonstrated in any enterococci isolated from 12 meat-processing plants.

Since 1989, vancomycin-resistant enterococci (VRE) have emerged as important nosocomial pathogens. In the United States, the prevalence of VRE in hospitals has increased from 0.3% in 1989 to 11%-13% by 1996 in patients other than those in intensive care units (1). Currently in the United States, more than half of all clinical isolates of *Enterococcus faecium* are not treatable with vancomycin (2). Another concern is that, as enterococci are found in the gastrointestinal tract of humans and animals, they may serve as a reservoir of glycopeptide resistance genes that may be transferred to other organisms such as methicillin-resistant *Staphylococcus aureus* and *S. epidermidis*.

The source of VRE is not known, although two potential reservoirs for these organisms are hospitals and food animals fed growth promoters, such as avoparcin (3,4). Padiglione et al. (5) found that human fecal colonization with VRE is uncommon in Australia, despite the relatively high level of consumption of avoparcin by industry (10,000 kg/year). In Europe, there have been numerous reports of VRE isolated from food animals and food products (4,6,7). In the United States, Knudtson and Hartman (8) studied the prevalence of antimicrobial resistance in enterococci from water, pork, and clinical isolates. In their studies, no VRE was detected from pork carcasses or fresh or spoiled pork products. Also in the United States, Coque et al. (9) and Thal et al. (10) studied enterococci from animal sources and did not recover VRE from the samples examined.

Avoparcin was licensed in Europe in 1975 and subsequently banned throughout the European Union in 1997 (6). In Australia, the per capita consumption of avoparcin by Australian agriculture is one of the highest in the world (5), while in the United States avoparcin has never been licensed for animal feed. The incidence of VRE infections in European countries is relatively low compared with the high, increasing rate in the United States, indicating that clinical use of vancomycin may be the reason for differences observed between the two populations.

The food chain has been proposed as a suspected source for dissemination of VRE to the human population (6). Should this be the case, one possible source is plants that produce ready-to-eat foods. Enterococci can be found in the environment of food-processing facilities, including those producing ready-to-eat meat and poultry products, but the incidence of vancomycin resistance among enterococci from these facilities is not known. We investigated the presence of VRE in 12 meat-processing plants in 8 U.S. states.

### The Study

A total of 446 swabs of floors and surfaces (in areas with and without food contact) were obtained from 12 meat-processing plants in 8 U.S. states during 1999. All processing facilities produced ready-to-eat meat products. The number of swabs collected at each plant ranged from 8 to 73. Swabs were taken by using either a sterile gauze pad or a sterile sponge moistened with Butterfield's phosphate diluent. The area covered by the swab depended on the sampling site. For sampling floors, an area of approximately 1 square meter was covered. All swabs were sent to the testing laboratory within 24 hours of collection. Swab samples were enriched in University of Vermont medium (UVM) and incubated at 30°C for 24 hours. Following enrichment, 0.1 mL of UVM culture was transferred to Fraser broth for selective enrichment and incubated at 35°C for 24 hours. All tubes that had visible growth or a darkened appearance due to esculin hydrolysis were streaked onto Bile Esculin Azide agar (BEAA) and incubated at 35°C for 48 hours. A single typical colony was selected from each sample and identified to genus level on the basis of esculin hydrolysis, bile tolerance, and colony morphology on BEAA. Enterococci were screened for vancomycin resistance by the method of Klein et al. (3) with some modifications. Enterococci isolates were subcultured onto Mueller-Hinton agar supplemented with 32 µg/mL vancomycin (MHV) to screen for high-level vancomycin resistance. Sensitivity of the isolate was determined by the confluence of bacterial growth on MHV plates after 48 hours of incubation at 35° C. No growth indicated sensitivity to vancomycin.

Address for correspondence: Bruce Tompkin, ConAgra Refrigerated Prepared Foods, Product Development Laboratory, 3131 Woodcreek Drive, Downers Grove, IL 60515, USA; fax: 630-512-1024; e-mail: btompkin@crfc.com

### Conclusions

The increasing prevalence of VRE in the United States is generally attributed to the hospital use of antibiotics (2,11). The meat-processing facilities examined in this study all produce ready-to-eat products made from beef, pork, or poultry. A total of 406 enterococci isolates from the plants' packaging areas were examined for high-level vancomycin resistance: 202 were isolated from food-contact areas and 134 from other areas and floors. No high-level vancomycin resistance was demonstrated in any enterococci isolated from the 12 meat-processing plant environments. These data suggest that enterococci with VanA resistance phenotype are uncommon in U.S. meat-processing facilities producing ready-to-eat meat products.

Dr. Bodnaruk was formerly employed by ConAgra Refrigerated Prepared Foods, Downers Grove, Illinois, as director of microbiology. He is currently microbiology section leader with Food Science Australia in Sydney. His major interests are innovative food-processing technologies and control of emerging food safety hazards.

### References

1. Robredo B, Singh KV, Baquero F, Murray BE, Torres C. From *vanA Enterococccus hirae* to *vanA Enterococcus faecium*: a study of feed supplementation with avoparcin and tylosin in young chickens. Antimicrob Agents Chemother 1999;43:1137-43.
2. Edwards DD. Enterococci attract attention of concerned microbiologists. ASM News 2000;66:540-5.
3. Klein G, Pack A, Reuter G. Antibiotic resistance patterns of enterococci and occurrence of vancomycin-resistant enterococci in raw minced beef and pork in Germany. Appl Environ Microbiol 1998;64:1825-30.
4. Wegener HC, Aarestrup FM, Jensen LB, Hammerum AM, Bager F. Use of antimicrobial growth promoters in food animals and *Enterococcus faecium* resistance to therapeutic antimicrobial drugs in Europe. Emerg Infect Dis 1999;5:329-35.
5. Padiglione A, Grabsch E, Olden D, Hellard M, Sinclair M, Fairley C, et al. Fecal colonization with vancomycin-resistant enterococci in Australia. Emerg Infect Dis 2000;6:534-6.
6. Robredo B, Singh KV, Baquero F, Murray BE, Torres C. Vancomycin-resistant enterococci isolated from animals and food. Int J Food Microbiol 2000;54:197-204.
7. Van Den Braak N, van Belkum A, van Keulen M, Vliegenthart J, Verbrugh HA, Endtz HP. Molecular characterization of vancomycin-resistant enterococci from hospitalized patients and poultry products in the Netherlands. J Clin Microbiol 1998;36:1927-32.
8. Knudtson LM, Hartman PA. Antibiotic resistance among enterococcal isolates from environmental and clinical sources. J Food Prot 1993;56:489-92.
9. Coque TM, Tomayko JF, Ricke SC, Okhyusen PC, Murray BE. Vancomycin-resistant enterococci from nosocomial community and animal sources in the United States. Antimicrob Agents Chemother 1996;40:2605-9.
10. Thal LA, Chow JW, Mahayni R, Bonilla H, Perri MB, Donabedian SA, et al. Characterization of antimicrobial resistance in enterococci of animal origin. Antimicrob Agents Chemother 1995;39:2112-5.
11. Davies R, Roberts TA. Antimicrobial susceptibility on enterococci recovered from commercial swine carcasses: effects of feed additives. Lett Appl Microbiol 1999;29:327-33.
